# First complete mitochondrial genome from the genus *Coptodryas* (Coleoptera: Curculionidae: Scolytinae) and its phylogenetic implications

**DOI:** 10.1080/23802359.2022.2055982

**Published:** 2022-04-01

**Authors:** Qiuhong Guo, Liyuan Liu, Feng Peng, Wen Sang, Xiaosheng Chen, Xingmin Wang

**Affiliations:** a Key Laboratory of Bio-Pesticide Innovation and Application, Engineering Technology Research Center of Agricultural Pest Biocontrol, Engineering Research Center of Biological Control, Ministry of Education, Guangzhou, China; b Department of Forest Protection, College of Forestry and Landscape Architecture, Guangdong Key Laboratory for Innovative Development and Utilization of Forest Plant Germplasm, South China Agricultural University, Guangzhou, China

**Keywords:** Mitochondrial genome, Curculionidae, Scolytinae, *Coptodryas elegans*, phylogenetic analysis

## Abstract

The complete mitochondrial genome (mitogenome) of *Coptodryas elegans* was determined, which represents the first sequenced mitogenome from *Coptodryas*. This mitogenome is 15,959 bp in size and comprises 36 typical coding genes and a control region, the *tRNA^Ile^* was not detected in this mitogenome, as observed in other species of Curculionidae. The monophyly of the family Scolytinae and the sister relationship between *C. elegans* and *Cyclorhipidion bodoanus* is supported by maximum likelihood analysis derived from the protein-coding gene sequences.

*Coptodryas* Hopkins, 1915 is a small genus, belonging to Xyleborini within Scolytinae, which is mainly distributed in tropical Asia and rare in Melanesia (Smith et al. 2020). Here, we described the complete mitogenome of *Coptodryas elegans* (Sampson, 1923), representing the first mitogenome from the genus *Coptodryas*, which will contribute to the molecular identification of this important pest and phylogenetic study of the Scolytinae.

The sample was collected from Chaozhou, Guangdong, China (N 23°85′11″ and E 116°63′26″) on 7 July 2019. Voucher specimen is stored at the Department of Entomology, South China Agricultural University (voucher no. X1, Xingmin Wang, wangxmcn@scau.edu.cn). Genomic DNA extraction was performed using the DNeasy Blood and tissue kit. An Illumina TruSeq library was prepared with an average insert size of 350 bp and sequenced using the Illumina Hiseq 2500 platform with 150 bp paired-end reads. High quality reads were used to produce a *de novo* assembly using IDBA-UD (Peng et al. 2012). Genomic annotations were performed using MITOS2 (Bernt et al. 2013) and tRNAscan-SE 2.0 (Lowe and Chan 2016), and the result was further confirmed using NCBI-BLAST (Figure 1).

**Figure 1.**
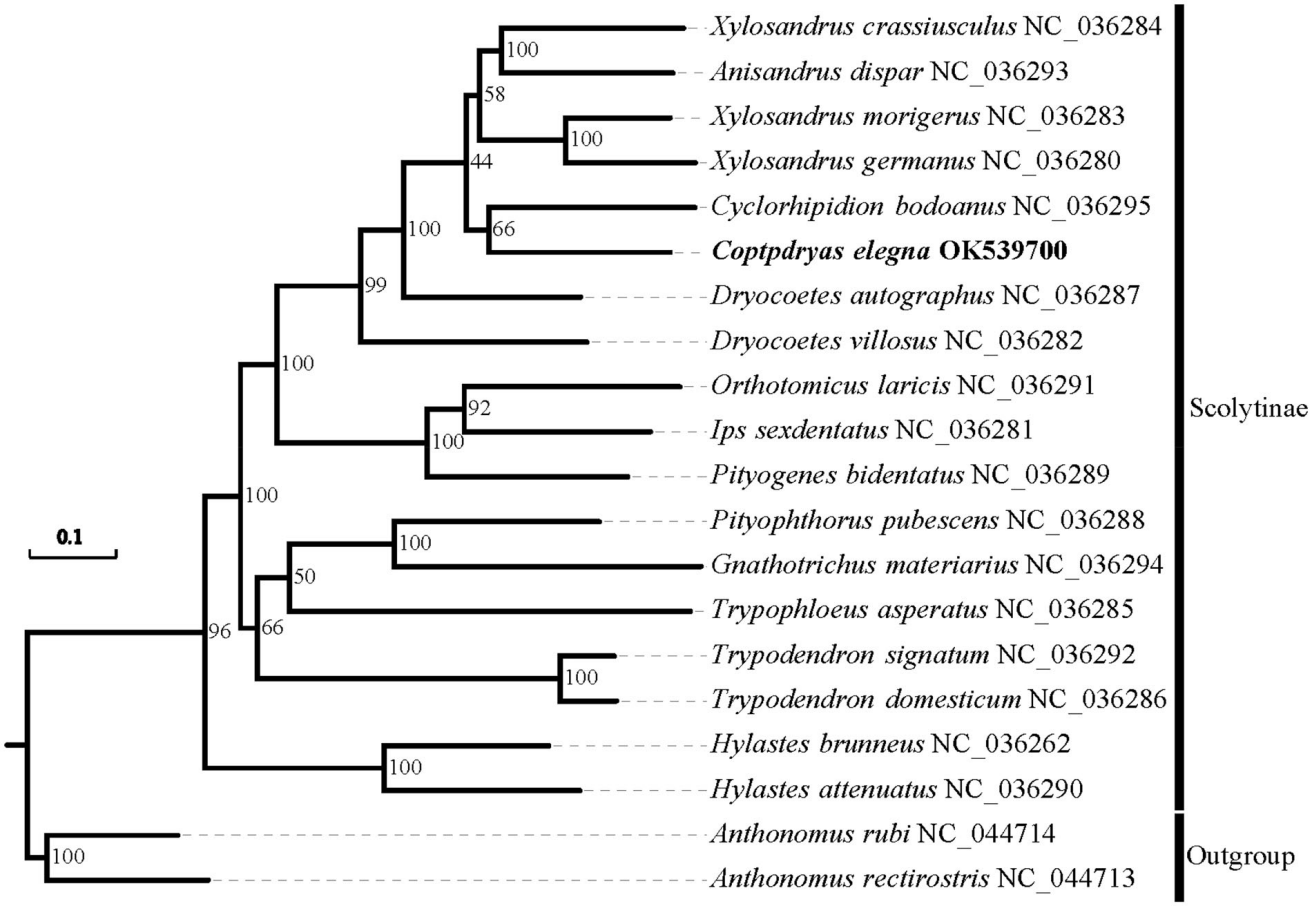
Phylogeny analysis of Scolytinae based on the concatenated nucleotide sequences of protein-coding genes. The newly sequenced *C. elegans* mitogenome is highlighted using bold and black.

The complete mitogenome of *C. elegans* is 15,959 bp in size, including 13 protein-coding genes (PCGs), 21 transfer RNA genes (tRNAs), two ribosomal RNA genes (rRNAs), and a control region. The *tRNA^Ile^* was not detected in the *C. elegans* mitogenome, as observed in other species of Curculionidae (Yang et al. 2018). The A + T content of the mitogenome is 73.7% (A = 40.7%, T = 33.1%, C = 16.7%, G = 9.5%) which is significantly biased toward AT. All PCGs initiate with ATN codons (four with ATA, four with ATG, three with ATT, and two with ATC). There are 21 tRNA genes, ranging from 63 to 72 bp in length. As in most other insect mitogenomes (Huang et al. 2021), the large and small rRNAs in *C. elegans* were located between *tRNA^Leu(CUN)^* and *^tRNAVal^* and between *tRNA^Val^* and the control region, respectively. The length of *lrRNA* and *srRNA* was determined to be 1301 bp and 783 bp, respectively.

Sequences of 13 PCGs from 18 Scolytinae species and two outgroup taxa from Curculioninae, were aligned by MAFFT 7.0 (Katoh and Standley 2013) and concatenated for phylogenetic analysis. Phylogenetic tree was inferred under the maximum-likelihood analysis using IQ-TREE 1.6.5 (Trifinopoulos et al. 2016). The monophyly of the Scolytinae is recovered with high supported (BS = 96) and the newly sequenced species *C. elegans* shows close relationship with *Cyclorhipidion bodoanus* (BS = 66).

## Data Availability

The data that support the findings of this study will be available in GenBank at https://www.ncbi.nlm.nih.gov/, accession number OK539700. The associated ‘BioProject’, ‘SRA’, and ‘Bio-Sample’ numbers are PRJNA808496, SRR18079461, and SAMN26088604, respectively.
